# *Corchorus Olitorius* Linn: A Rich Source of Ω3-Fatty Acids

**DOI:** 10.4172/2153-2435.1000486

**Published:** 2016-06-28

**Authors:** AS Mahmoud, N Thao, A Mario

**Affiliations:** 1 Department of Chemistry, Texas Southern University, 3100 Cleburne Street, Houston, TX 77004, USA; 2 Agilent Technologies, Inc., 3750 Brookside Parkway, Suite 100, Alpharetta, GA 30022, USA

**Keywords:** Molokhia, GCQTOF, Chemical ionization, Electron ionization, Fatty acids methyl esters, FAMES

## Abstract

Fatty acids composition of *Corchorus olitorius* Linn were identified as their methyl esters using accurate mass gas chromatography quadrupole time of flight mass spectrometry (GCQTOF) in chemical ionization (CI) and electron ionization (EI) modes. The leaves which are the edible part of the plant were found to be very rich in ω3-octadecatriene fatty acid reaching up to more than 49 % of the total fatty acids contents. This is the first report to unequivocally detect ω-3 fatty acid in *Corchorus olitorius* Linn with a much higher concentration than any other reported vegetable and further investigation into its health effects are clearly warranted.

## Introduction

*Corchorus olitorius* of the Tiliaceae family is an annual, unbranched herb, 90 to 120 cm tall, with glabrous stems, leaves 6 to 10 cm long and 3.5 to 5 cm broad, with pale yellow flowers and black trigonous seeds [1]. It grows wild in Africa, Egypt, Middle East, Philippines, Thailand, India and Nepal [2–6]. The plant is cultivated in Egypt for the leaves, which provide one of the most popular potherb known as Molokhia [1]. India, Pakistan and Bangladesh cultivate the plant for its fiber and as a folk medicine [7]. For Egyptians, Molokhia has been the symbol of their homeland for a long period of time. It was shown to have demulcent, diuretic, lactagogue, purgative and tonic properties and is used as a folk remedy for aches, pains and swellings [8,9]. Phytochemical constituents of *Corchorus olitorius* including bioactive molecules, proteins, carbohydrates and terpenoides were reported in the literature, but little is known about its lipids and fatty acids composition [10,11]. In this manuscript we used accurate mass GCQTOF to identify and quantify its fatty acids composition.

## Methods

### Reagents and standards

All general use solvents and chemical reagents were purchased from VWR (Sugar Land, TX, USA). Iso-octane and 10% Boron trifluoride in methanol were obtained from Sigma-Aldrich (Milwaukee, WI, USA). Complete set of fatty acids methyl esters both saturated and unsaturated from C4 to C26 were obtained from Sigma-Aldrich (USA) and were used for GC retention time comparison. The rest of the reagents were all of analytical grade.

### Plant materials

Two packages of 10 pounds each of authentic dry leaves were obtained from Phoenicia Specialty Foods (Houston, TX, United States). One sample was imported from Egypt and the other was imported from Lebanon.

### Sample preparation

Dry leaves were homogenized, grounded to fine powder and aliquots were extracted under Argon in the dark using ultrasound assisted extraction using Qsonica Sonicators (Model Q700, CT, USA) with a working frequency of 20 KHz and a maximum input power up to 700 W. 10 g of each sample and 30 mL of chloroform/methanol (2:1 v: v) were sonicated at room temperature with amplitude mode at 50; power at 17 W; energy at 4308 J; process time: 5 min and repeated 3 times for a total extraction time of 15 minutes. Extracts were filtered, dried over anhydrous sodium sulfate. Solvent was removed by vacuum distillation using rotary evaporator at a temperature below 40 °C, stored under argon at −80 °C until further use.

### Fatty acids methyl esters (FAMES) preparation

FAMES preparation was performed as we described before [12] using 10 mg of the neat crude lipid extract which was added to 2 mL of 10% BF_3_ in methanol, mixed by vortex and placed on the heating block at 75 °C for 1 h. After cooling down to room temperature, 1 mL of saturated sodium chloride solution was added to stop the reaction and FAMES were extracted in 2 mL of iso-octane, passed through anhydrous sodium sulfate and transferred to GC auto-sampler vials.

### GCQTOF analysis

Agilent 7200 accurate mass GCQTOF System was used for analyzing FAMES samples. GC separation was done using a BPX90 SGE Analytical Science column 15 m × 0.25 mm × 0.25µm column (SGE Analytical Science, Texas) run under average velocity of 63.9 cm/sec with a hold up time of 1 min. Temperature programming started at 75°C held for 2 minutes and heated up to 200 °C at a rate of 5 °C/min, held at 200°C for 3 min. Total run time was 30 minutes; Solvent delay 4 min; equilibration time 3 min. He collision gas 1.5 mL/min; injected volume 1µL; split mode 10:1. Mass spectral acquisition were separately performed in EI mode and CI mode using methane as the reagent gas. The measurements and post-run analyses were controlled by the software Mass Hunter Qualitative Analysis B.07 [13].

### GCQTOF MS-MS

MSMS experiments were conducted at 25 ev, run time 30 min; ion source: chemical ionization; source gas temperature 300°C as we previously described [13]. He collision gas 1.5 mL/min; ms/ms mass range 50 m/z to 300 m/z; acquisition rate 5.0 spectrum/s; acquisition time 500 ms/spectrum; 6610transients/ spectrum; auto recalibration reference mass window, detection window 100 ppm; minimum height 1000 counts; polarity type: positive.

## Results and discussion

Crude lipids extracts were obtained from dry leaves using ultrasound assisted extraction as shown in the experimental sections. Yield (w/w) was 4.8% and 4.5% for Egyptian and Lebanon samples respectively.

### Fatty acids composition

**Figure 1:**
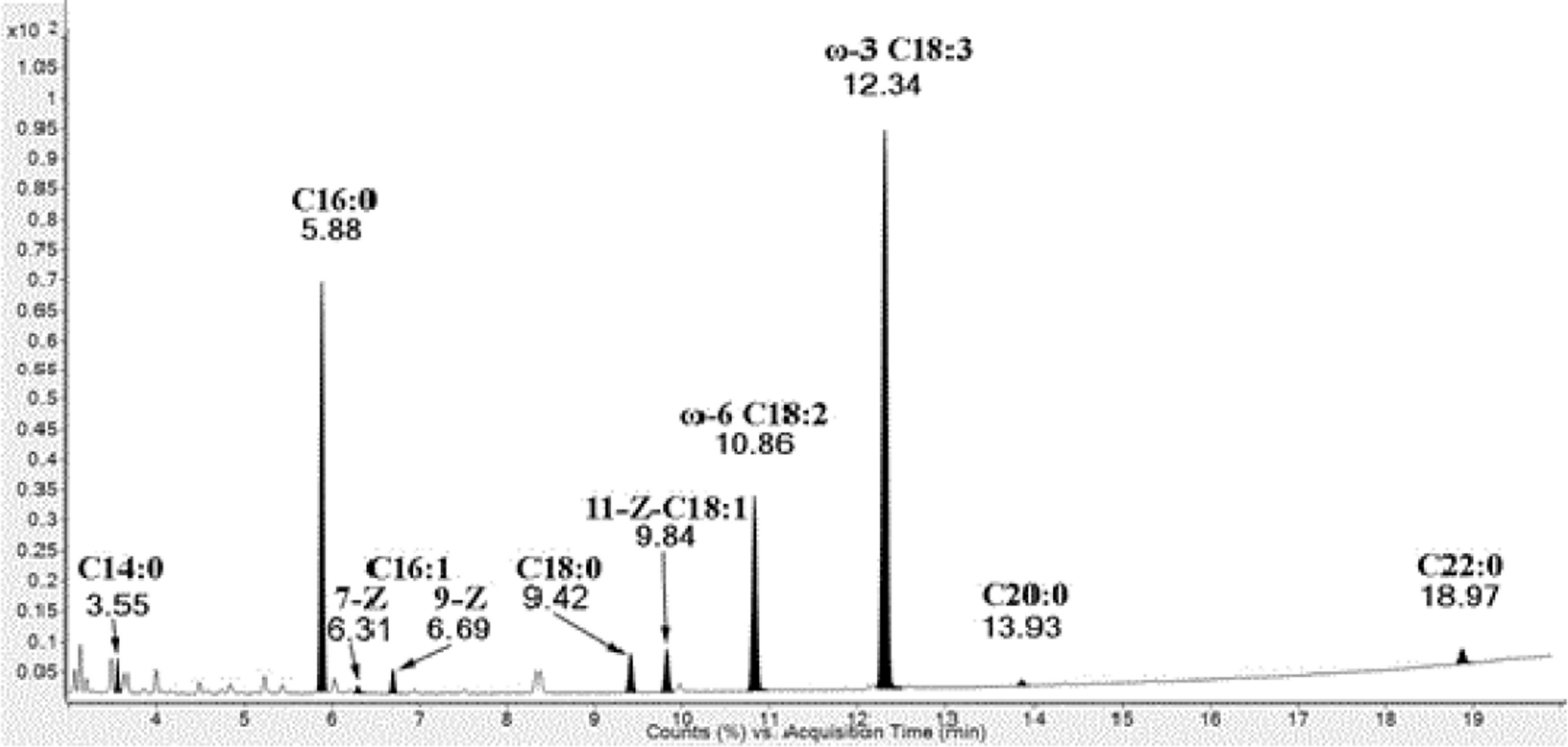
TIC of GCQTOF analysis of fatty acids methyl esters of *Corchorus olitorius*. Peaks that are not highlighted are nonfatty acids and are mostly hydrocarbons, fatty alcohols and terpenoides.

Fatty acid methyl esters obtained from dry leaves were analyzed using accurate mass GCQTOF mass spectrometry. Relative percentage composition of fatty acids methyl esters (FAMES) in each oil is shown in Table 1. FAMES were identified based on their retention times and their accurate mass data. Their electron ionization fragmentation and mass spectral data were also searched using Wiley10NIST mass spectral database. GCMS analysis of the fatty acids methyl esters (FAMES) was achieved with base line resolution as shown in Figure 1. Additionally, identification of ω3-octadecatriene fatty acid methyl ester (mw of 292.2380) was confirmed by ms-ms experiment. The results showed that dry leaves are extremely rich in ω-3 fatty acid with a relative concentration of 49.5% in the Egyptian samples and 46.23 % in the Lebanon samples. The second most popular fatty acid was palmitic acid (C16:0) with a relative percentage of about 23% in both Egyptian and Lebanese samples. Stearic acid (C18:0) was less than 4% in all samples.

**Figure 2:**
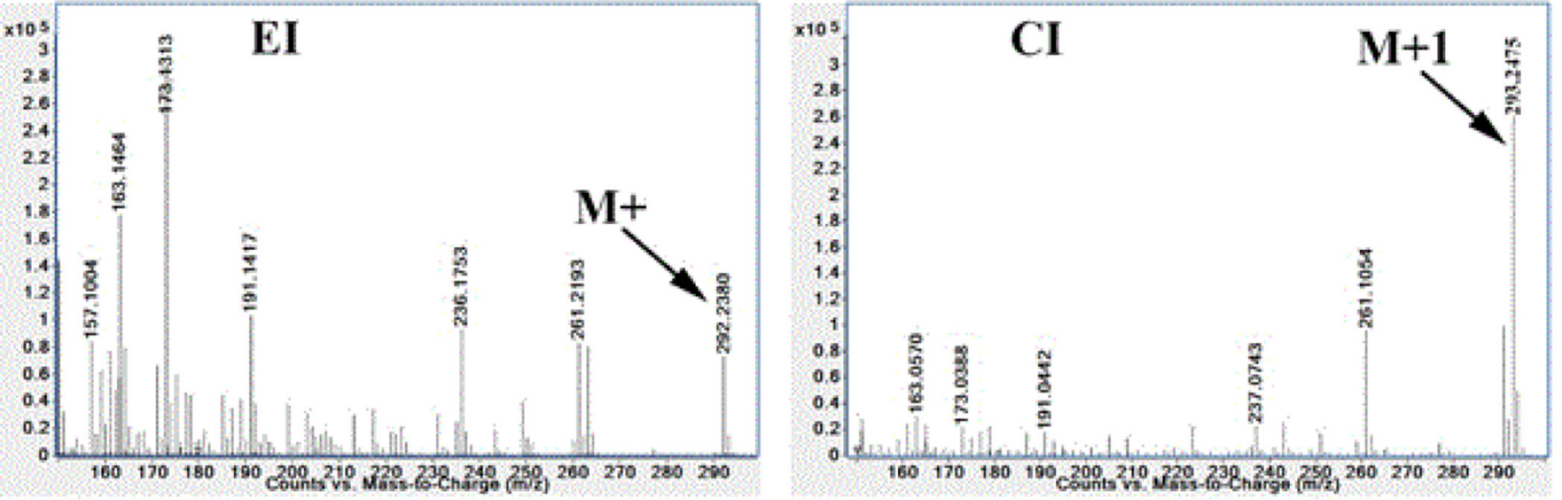
Mass spectra of ω-3 C18:3 fatty acid, EI left and CI right.

Accurate mass chemical ionization provided information about the intact molecular ion as an (M+1)^+^ ion as the base peak with much less fragmentations. Electron ionization provided fragments that can be used to predict the structure but provide much less abundance of the molecular ion. Conformation of the presence of ω-3 fatty acids was carried out by comparing retention time to authentic standards and by measuring accurate mass in CI mode in addition to examining fragmentation pattern in the EI mode as shown Figure 2.

**Figure 3:**
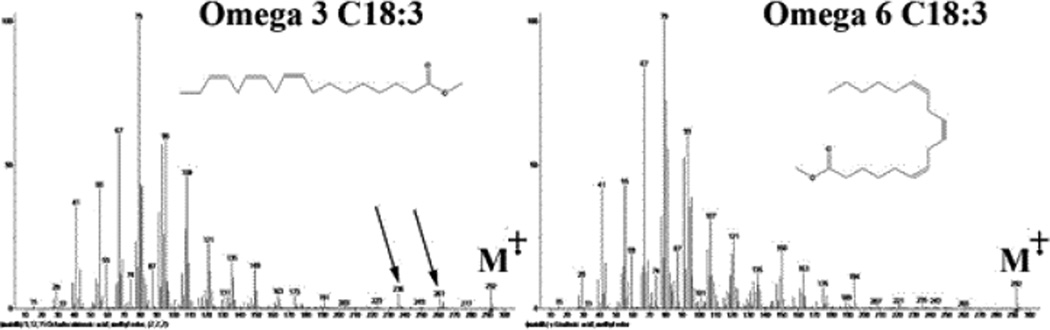
Mass spectra of ω-3 C18:3 (left) and ω-6 C18:3 (right) fatty acids.

Additionally, ω-3 fatty acids can be differentiated from ω-6 fatty acids by the higher abundance of (M-CH_3_)+ corresponding to m/z of 261 and another ion at a m/z of 236 corresponding to the loss of C_4_H_8_ radical (CH_3_CH_2_CH=CH_2_) for the ω-3 C18:3 fatty acids. The loss of a methyl radical to the ion at m/z of 261 is favorable for of ω-3 due to formation of an allylic carbocation, while in ω-6 fatty acids will form a primary carbocation. Also the loss of C_4_H_8_ radical cannot take place in ω-3 fatty acids due to the absence of a double bond at the of ω-3 position. This was also confirmed both by comparing mass spectral fragmentation for ω-3 and ω-6 fatty acids as shown in Figure 3 where ω-6 did not show any of the two ions. Further confirmation was carried out by the MSMS experiment where the two fragments were detected in ω-3 but not ω-6.

Polyunsaturated fatty acids are essential in the human diet since they cannot be synthesized by the body [14]. The essential fatty acids are very important to human immune system, to help regulate blood pressure. The ω-3 and ω-6 fatty acid are found in some food; fish, shellfish, flax seed, soya oil, canola oil, hemp oil, chia seed, pumpkin seed, sunflower seed, cotton seed oil, leafy vegetables and walnuts [15,16]. This is the first report to unequivocally detect ω-3 fatty acid in *Corchorus olitorius* with a much higher concentration than any other reported vegetable. A number of animal experiments, epidemiological investigations have confirmed the essentiality of ω-3 fatty acids for normal retina and brain development of the premature infant, and for its hypotriglyceridemic, anti-inflammatory, and antithrombotic properties [15,16]. Most of the studies have been carried out with fish oils (eicosapentaenoic acid (EPA) and DHA). However, ω-3 C18:3 (alpha- linolenic acid), found in green leafy vegetables, flaxseed, rapeseed and walnuts, desaturates and elongates in the human body to EPA and DHA and by itself may have beneficial effects in health and in the control of chronic diseases [17].

## Conclusion

Corchorus olitorius Linn a traditional vegetable in Egypt and throughout the Middle East was found to be very rich in the essential fatty acid ω-3 C18:3 with more than 40% of the total fatty acids.

## Figures and Tables

**Table 1:** FAMES composition of *Corchorus olitorius*

Retention Time (min)	Identified compounds Fatty acids methyl esters	Egyptian Samples	Lebanese Samples
3.55	Tetradecanoic acid (C14:0)	1.37 ± 0.01	1.19 ± 0.01
5.88	Hexadecanoic acid (C16:0)	23.36 ± 0.60	23.08 ± 1.48
6.31	7-Z-Hexadecenoic acid (C16:1)	0.53 ± 0.15	0.50 ± 0.02
6.69	Palmitoleic acid (C16:1) 9-Z	1.36 ± 0.01	1.79 ± 0.02
9.42	Stearic acid C18:0	2.68 ± 0.00	3.10 ± 0.04
9.84	11-Z-Octadecenoic acid C18:1	3.12 ± 0.06	4.29 ± 0.08
10.86	9,12-ZZ- Octadecadienoic acid ω-6 C18:2	15.18 ± 0.55	17.94 ± 1.75
12.34	9,12,15-(Z,Z,Z)- Octadecatrienoic acid ω-3 C18:3	49.50 ± 2.53	46.23 ± 0.84
13.93	Eicosanoic acid C20:0	0.54 ± 0.00	0.66 ± 0.01
18.97	Docosanoic acid C22:0	1.44 ± 0.01	1.21 ± 0.01
